# Wound Dressings Based on Sodium Alginate–Polyvinyl Alcohol–*Moringa oleifera* Extracts

**DOI:** 10.3390/pharmaceutics15041270

**Published:** 2023-04-18

**Authors:** Samir Kamel, Sawsan Dacrory, Peter Hesemann, Nadir Bettache, Lamiaa M. A. Ali, Lou Postel, Engy M. Akl, Mohamed El-Sakhawy

**Affiliations:** 1 Cellulose & Paper Department, National Research Centre, 33 El Bohouth St., Dokki, Giza 12622, Egypt; 2 ICGM, Université Montpellier, CNRS, ENSCM, CEDEX 05, 34095 Montpellier, France; 3 IBMM, Université Montpellier, CNRS, ENSCM, 34093 Montpellier, France; 4 Department of Biochemistry, Medical Research Institute, Alexandria University, Alexandria 21561, Egypt; 5 Fats and Oils Department, Food Industry and Nutrition, National Research Centre, 33 El Bohouth St., Dokki, Giza 12622, Egypt

**Keywords:** poly(vinyl)alcohol, sodium alginate, *Moringa oleifera* leaves, cytocompatibility, wound healing, biopolymers, polyphenolic, fibroblasts

## Abstract

Biopolymers have significant pharmaceutical applications, and their blending has favorable characteristics for their pharmaceutical properties compared to the sole components. In this work, sodium alginate (SA) as a marine biopolymer was blended with poly(vinyl) alcohol (PVA) to form SA/PVA scaffolds through the freeze–thawing technique. Additionally, polyphenolic compounds in *Moringa oleifera* leaves were extracted by different solvents, and it was found that extracts with 80% methanol had the highest antioxidant activity. Different concentrations (0.0–2.5%) of this extract were successfully immobilized in SA/PVA scaffolds during preparation. The characterization of the scaffolds was carried out via FT-IR, XRD, TG, and SEM. The pure and *Moringa oleifera* extract immobilized SA/PVA scaffolds (MOE/SA/PVA) showed high biocompatibility with human fibroblasts. Further, they showed excellent in vitro and in vivo wound healing capacity, with the best effect noted for the scaffold with high extract content (2.5%).

## 1. Introduction

*Moringa oleifera* is a fast-growing drought-resistant tree belonging to the Moringaceae (mono-generic) genus. This plant is found in a wild form as well as a cultivated form in the plains, and its height varies from 5 to 10 m [1]. It is known as the ‘Miracle Tree’, owing to its medicinal and nutritional values. Seeds, flowers, fruits, leaves, roots, and immature pods are nontoxic and contain minerals, nutrients, and various antioxidants that are consumed as food in tropical countries, and also used in traditional medicine to treat multiple diseases without side effects. The leaf extracts of Moringa and the gum exudates from its stem have been studied for their wound-healing properties [1], and for their film-forming and gelling abilities [2], respectively. Additionally, the phytochemical analysis of the seed shows all the essential constituents necessary for efficient wound healing activity [3].

*Moringa oleifera* leaves have phenolic compounds such as gallic acid and flavonoids such as kaempferol and quercetin associated with the antioxidant compounds responsible for scavenging free radicals. Antioxidants protect tissues against cellular damage by stabilizing free oxygen molecules [4]. Hossain et al. proved that the methanolic leaf extract of *Moringa oleifera* inhibited human breast cancer (MCF-7) cell growth [5].

The utilization of natural polysaccharides in wound healing continues to be a subject of intense research owing to their biodegradability and biocompatibility [6]. One of these biosourced materials is sodium alginate (SA), which presents interesting properties such as high protein absorption ability, fast biodegradability, high hydrophilicity, antibacterial activity, and satisfactory hemostatic and biological properties [7,8]. SA, as a component of different mixtures, has been used to modify the wound dressing properties of poly(vinyl)alcohol (PVA) hydrogels [9].

One of the few synthetic biodegradable polymers is PVA, which has satisfactory properties such as nontoxicity, biocompatibility, water solubility, and good film-forming ability; besides these, it is a cost-effective, ductile, and flexible material. Moreover, PVA has good mechanical properties that support cell adhesion, propagation, and migration, making it suitable for biomedical applications, particularly wound dressing fabrication [10]. The presence of hydroxyl groups on the PVA backbone facilitates the formation of hydrogen bonds with different biopolymers. Therefore, increasing attention has been focused on designing environmentally compatible PVA-based materials for wound dressing applications. However, PVA is inertly bioactive and cannot be administrated as a bio-functional wound dressing for complex healing wounds. Thus, for producing bioactive PVA-based wound dressings, it is blended with other biomaterials and/or bioactive molecules to exhibit better cellular interaction that, in turn, accelerates wound healing [11].

Three-dimensional hydrophilic networks, such as hydrogels containing >70% water, promote successful rehydration. They have multiple advantages, such as supporting immediate pain control, their easy replacement, absorbance capacity, preventing the loss of body fluids, acting as a barrier to invading microbes, and permitting gaseous exchange. Additionally, they provide a moist environment for the wound area [12]. Hydrogels based on natural polysaccharides are suitable candidates for wound dressing applications due to their biocompatibility, biodegradability, and fluid absorption properties, along with their easy synthesis and processability [13]. Furthermore, adding polysaccharides to the polymer matrix also improves the swelling of the hydrogel wound dressings to avoid a repeated change of dressing [14,15]. Hydrogel wound dressings are the primary therapeutic strategy for all types of wound treatment to restore homeostasis and manage skin repair. Various hydrogels based on natural polysaccharides and synthetic polymers have recently been reported for drug-controlled release and wound dressing applications [16,17].

Studies have used SA/PVA as a scaffold with and without synthetic material to enhance its activity [7,18,19]. PVA-loaded *Moringa oleifera* seed extract has been synthesized as a wound dressing material to help heal chronic wounds [20]. In another work, PVA-loaded *Moringa oleifera* leaf extract with graphene oxide hydrogel was developed as a wound dressing by Ningrum et al. for wound dressing [21]. Additionally, SA/PVA/chitosan hydrogel with Ag_2_O/SiO_2_ and *Calendula officinalis* flower extract have been fabricated as a novel and green wound dressing [22].

Accordingly, this study aims to prepare a new hydrogel by immobilizing polyphenol extracted from the *Moringa oleifera* leaves onto a scaffold based on SA and PVA prepared by the freeze–thawing cycle technique. Firstly, the polyphenolic compounds of *Moringa oleifera* leaves were extracted using different solvents. Then, the antioxidant activity was assessed by the 2,2-Diphenyl-1-picrylhydrazyl (DPPH) free radical scavenging assay for all extracts. The extract with the highest scavenging activity was selected and immobilized with different concentrations (0.0–2.5%) into the SA/PVA scaffolds. After, their cytotoxicity and wound healing abilities were assessed.

## 2. Materials and Methods

### 2.1. Materials

*Moringa oleifera* leaves were collected from a farm in Ismalia, Egypt. The leaves were rinsed with tap water, dried in a thermostatic drying oven at 70 °C, and then crushed into a fine powder using a mortar and pestle. Sodium Alginate and poly(vinyl)alcohol were purchased from Sigma Aldrich. Folin–Ciocalteu’s phenol reagent and 2,2-Diphenyl-1-picrylhydrazyl (DPPH) were purchased from Sigma-Aldrich. MTT (4,5-dimethylthiazol-2-yl)-2,5-diphenyltetrazolium bromide) was provided by ThermoFisher Scientific (Courtaboeuf, France). All chemical reagents were used as received.

### 2.2. Methods

#### 2.2.1. Chemical Composition of *Moringa oleifera* leaves

Moisture, protein, oil, ash, and fiber were determined according to AOCS 2005 standard analysis methods, and the nitrogen-free extract was calculated.

#### 2.2.2. Extraction of Phenolic and Flavonoid Compounds

One gram of powdered leaves was subjected to 50 mL of extraction solvent (0.1 N potassium persulfate, 0.25 N NaOH, 0.25 N HCl, 80% Methanol, 80% Ethanol, 80% Ethylacetate, or 80% Acetone) and set in an ultrasonic bath at about 50 °C for 1 h and then centrifuged for 20 min at 3000 rpm, giving supernatant A. Next, the residue was macerated in another quantity of solvent. Then, the first process was repeated three times to provide supernatants B, C, and D. Collected supernatants were used to determine the total phenolic and flavonoid contents and estimated their antioxidant capacity [23].

#### 2.2.3. Determination of Total Phenolic (TPC) and Flavonoids (TFC) Compounds

The content of phenolic and flavonoid compounds was determined according to the method described in the literature [23,24]. First, 200 μL of the sample was made up to 3 mL with distilled water, then 2 mL of 10% folin reagent was added and sample was shaken for 5 min. Next, 1 mL of 7.5% sodium carbonate was added and left for one hour in the dark. Finally, the absorbance at 765 and 510 nm was measured using a spectrophotometer (T80 UV–Vis spectrophotometer) for the determination of TPC and TFC, respectively.

The sample analysis was achieved using liquid chromatography-electrospray ionization–tandem mass spectrometry (LC-ESI-MS/MS) with an Exion LC AC system for separation and a SCIEX Triple Quad 5500 + MS/MS system supplied with electrospray ionization (ESI) for detection.

The separation was performed using a ZORBAX SB-C18 Column (4.6 × 100 mm, 1.8 µm). The mobile phases contained two eluents: A, 0.1% formic acid in water, and B; acetonitrile (LC grade). The mobile phase was planned as follows: 2% B from 0 to 1 min, 2–60% B from 1 to 21 min, 60% B from 21 to 25 min, and 2% B from 25 to 28 min. The flow rate was 0.8 mL/min, and the injection volume was 3 µL. For the MRM analysis of the selected polyphenols, positive and negative ionization modes were applied in the same run with the following parameters—curtain gas: 25 psi; IonSpray voltage: 4500 and 4500 for positive and negative modes, respectively; source temperature: 400 °C; ion source gas 1 and 2 were 55 psi with a decluttering potential: 50; collision energy: 25; and collision energy spread: 10.

#### 2.2.4. Antioxidant Activity

The DPPH scavenging assay, which is commonly known as an easy and rapid test for assessing the presence of antioxidants and for its ability to scavenge the oxidative stress-producing free radicals in a sample, was completed. The scavenging activity of DPPH free radicals was measured according to the method described by Zhao and coworkers [25].

First, the DPPH solution was prepared by adding 4 mg of DPPH to 100 mL methanol (it produced a violet color). Then, 4 mL of the solution and different volumes of *Moringa oleifera* leaves extract (10, 20, 40, 80, and 100 µL) were mixed, shaken vigorously, and left in the dark to stand at 30 °C for 30 min. 

The decolorization of the methanolic DPPH solution was determined by measuring the decrease in absorbance at 517 nm using a spectrophotometer model (UV VIS Spectrophotometer PG Instruments United Kingdom). Results were expressed as an inhibition percentage of the DPPH using the following equation [26].

(1) $$\mathrm{Inhibition}\;\%=\frac{\mathrm{Absorbance}\;\mathrm{Control}-\;\mathrm{Absorbance}\;\mathrm{Sample}}{\mathrm{Absorbance}\;\mathrm{Control}\;}\times 100$$

The absorbance of control is the absorbance of the DPPH solution without extract.

In addition, the concentration of IC_50_, the level at which 50% of radicals was scavenged by test or standard sample, was measured from the calibration curve. All the experiments were performed in triplicate.

The IC50 values can be calculated in the EXCEL program by plotting the curve of inhibitions and corresponding concentrations.

#### 2.2.5. Preparation of Scaffolds

**Scheme 1:**
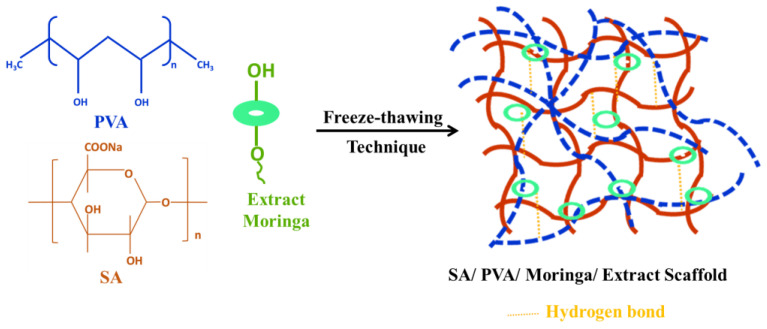
Schematic diagram of chemical bonding between SA, PVA, and MOE.

A scaffold composed of sodium alginate (SA), *Moringa oleifera* extract (MOE), and poly(vinyl)alcohol (PVA) was prepared by a freezing-thawing cycle (Scheme 1). Briefly, an aqueous solution of PVA (2 g), SA (1 g) and different proportions of MOE (0, 1, 1.5, 2, and 2.5 mL) were mixed in 100 mL distilled water and sonicated in the water bath at 30 °C for 1 h. Then, the mixtures were poured into Petri dishes, followed by freezing for 6 h and thawing for 6 h at 25 °C for six continuous cycles to provide acceptable hydrogels. Finally, the attained scaffolds were lyophilized for further investigations and coded as S1, S2, S3, S4, and S5, respectively.

#### 2.2.6. Scaffold Characterization Methods

FT-IR spectra were measured in the range of 400–4000 cm^−1^ on the FT-IR Spectrophotometer (Shimadzu 8400S).

Diano X-ray diffractometer using a CoKα radiation source energized at 45 kV and a Philips X-ray diffractometer (PW 1930 generator, PW 1820 goniometer) with a CuK radiation source (λ = 0.15418 nm), at a diffraction angle range of 2θ from 10 to 70° in reflection mode, was used to investigate the XRD patterns of the samples.

Thermogravimetric analyses (TGA) were carried out using a TGA Perkin-Elmer (STA6000), with a heating rate of 10 °C/min. The temperature ranged from room temperature up to 900 °C under air atmosphere.

The morphology of the hydrogels was studied by freeze-drying of hydrogels, and they were gently cut to obtain a smooth surface. A Hitachi S-4800 scanning electron microscope was used to visualize the surface morphology of the samples. Prior to the SEM examination, the samples were coated with gold using a vacuum sputter coater.

#### 2.2.7. Cell Line

Human dermal fibroblasts were purchased from Lifeline^®^ Cell Technology (Frederick, MD, USA) and maintained in Roswell Park Memorial Institute (RPMI) medium supplemented with 10% fetal bovine serum (FBS) and 1% penicillin/streptomycin (P/S).

#### 2.2.8. Cell Viability (MTT) Assay

Fibroblasts were seeded in a 96-well plate at a density of 5000 cells *per* well and left to incubate for 24 h. Before treatments, pure extract and MOE/SA/PVA suspensions were prepared in water containing P/S followed by sonication, and then cells were treated with different concentrations of these suspensions, ranging from 0 to 400 µg mL^−1^. Control cells were treated with the vehicle. After an incubation of 3 days, we added the MTT solution to cells at a final concentration of 0.5 mg mL^−1^ and cells were incubated for 4 h at 37 °C. After the end of incubation, the medium was aspirated and the formed violet crystals were dissolved in a mixture of ethanol and DMSO (1:1, *v*/*v* %), followed by 20 min shaking. The absorbance was measured using multiskan Sky (ThermoFisher scientific) at 540 nm and the percentage (%) of viable cells was calculated from the following equation: Ab_test_/Ab_control_ × 100.

#### 2.2.9. In Vitro Wound Healing Assay

Fibroblasts were plated at 100% confluency on 24-well plates. After washing with PBS, a confluent area was then scratched with a pipette tip, and then cells were cultured in a culture medium containing different concentrations of pure extract and MOE/SA/PVA. The same volume of a complete medium was added as vehicle. The cell migration into the gap was followed by microscopy imaging at 0 and 24 h after different treatments. At each time point, images were acquired using an EVOS™ M5000 Imaging System (ThermoFisher Scientific, Waltham, MA, USA) with a 4× objective.

#### 2.2.10. In Vivo Wound Healing Assay

Casper zebrafish embryos were obtained from the lab’s facilities of molecular mechanisms in neurodegenerative dementia (MMDN), Inserm U1198, Montpellier University, Montpellier. Embryos were maintained in Petri dishes with a sufficient amount of water at 28 °C and a 14 h light/10 h dark cycle. At 72 h post fertilization (hpf), the caudal fin was transected (or not) with a sterile scalpel, under anesthesia with 168 µg/mL of Tricaine (ethyl 3-aminobenzoate, Sigma Aldrich, France) in zebrafish water, and imaged using EVOS M5000 microscopy (T0). Embryos were kept in 24-well plates, one embryo per well, with 2 mL of water containing 0 or 1 mg/mL or 2 mg/mL of *Moringa oleifera* extract immobilized SA/PVA scaffold suspensions (S5). Embryos were observed after 3 days of treatments (T72). The area of the tail of each embryo was calculated using ImageJ software at T0 and T72. The growth percentage in each embryo was calculated as T72/T0 × 100. Results are presented as mean ± SEM. The number of embryos was 10 for each condition.

#### 2.2.11. Statistical Analysis

All experiments were performed in triplicate, and values were expressed as mean ± standard deviation (SD). Significant statistical differences in the explored parameters were determined and analyzed using one-way analysis of variance (ANOVA PC-STAT, 1985 VERSION IA copyright, University of Georgia). A confidence interval at the 95% level and a probability (*p*) value of less than 0.05 were considered statistically significant at the 5% level of significance (*p* < 0.05).

## 3. Results and Discussion

### 3.1. Analysis and Activities of Moringa Oleifera Leaves Extract

**Figure 1:**
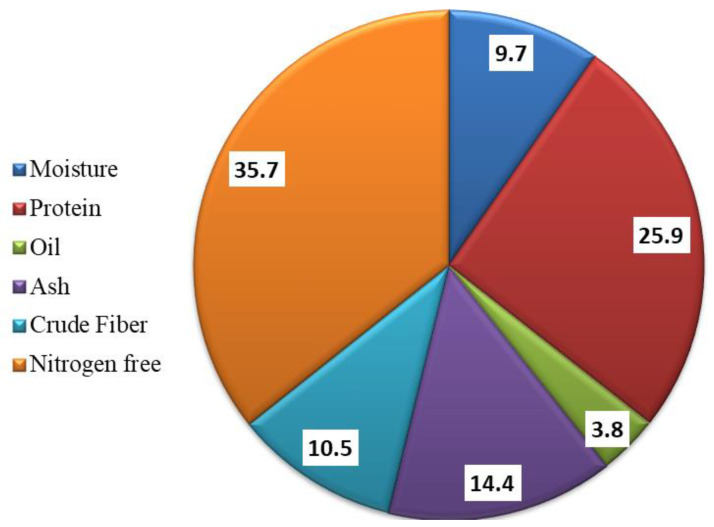
Composition of *Moringa oleifera* leaves.

Our study aimed to evaluate the antioxidant biocompatibility and wound healing potential of *Moringa oleifera*-leaf-extract-immobilized SA/PVA scaffold. *Moringa oleifera* leaves are considered to have high contents of nutrients in the plant, such as proteins, essential amino acids, and minerals. Figure 1 represents the chemical composition of *Moringa oleifera* leaves. It shows that the protein content was about 25.9%, the oil content was about 3.8%, and they had high ash content. Previous studies have showed that *Moringa oleifera* has intense antioxidant activity due to the presence of vitamins, phenolics, and flavonoids [27,28].

Table 1 represents the TPC and TFC and the antioxidant capacity of *Moringa oleifera* leaves extracts, obtained with different solvents. The highest TPC values were 334.3 ± 2 mg/g, 219.2 ± 1.6 mg/g, and 197.1 ± 1.5 mg/g for the extracts obtained with aqueous sodium hydroxide (0.25 N), methanol, and acetone, respectively. The high content of phenolic compounds of 0.25 N NaOH as an extraction solvent was attributed to a high content of proteins, carbohydrates, polysaccharides, electrolytes, and other hydrophilic nutrients, which are sufficiently soluble in basic media [29]. In contrast, the highest TFC values were 34.9 ± 0.6 mg/g, 19.9 ± 0.5 mg/g, and 13.7 ± 0.4 mg/g, obtained via extraction with acetone, ethanol, and methanol, respectively. The lowest TPC and TFC were obtained in the case of extraction by K_2_S_2_O_8_ and HCl. In general, the TPC was higher than TFC, which may be attributed to the higher solubility of phenolic compounds in solvents than flavonoids.

The IC_50_ of the scavenging activity was determined for all extracts by the DPPH method, which is the minimum amount that allows 50% scavenging to the free radical of DPPH. Methanol and hydrochloric acid 0.25 N extracts had the least IC_50_ among all treatments; however, the methanol extracts exhibited a larger amount of TPC and TFC than hydrochloric acid 0.25 N extracts.

Free radicals are produced by the phagocytes when an inflammation-causing wound occurs. One of the beneficial therapeutic strategies in wound healing could be their inhibition by the process of scavenging free radicals, since an increase in free radical production delays the healing process.

*Moringa oleifera* leaves contain phytochemical compounds such as tannins, saponins, flavonoids, carbohydrates, and polyphenolic compounds [30]. Considering the results of DPPH IC_50_, 80% methanol was selected as an extraction solvent in the next stage for further immobilization onto the SA/PVA scaffold. The polyphenolic compounds of the methanol extract were determined using liquid chromatography-electrospray ionization–tandem mass spectrometry (LC-ESI-MS/MS). In this way, chlorogenic acid, coumaric, caffeic, naringenin, 3,4-Dihydroxybenzoic acid, Cinnamic acid, and other components could be identified (Table 2).

### 3.2. Characterization of Scaffolds

#### 3.2.1. FT-IR Spectra

**Figure 2:**
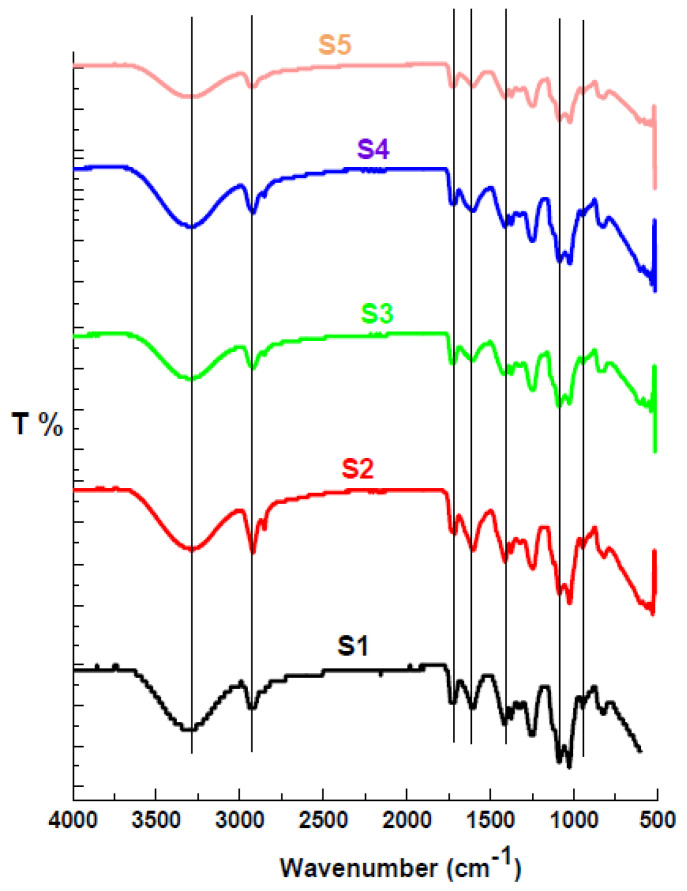
FTIR spectra of the MOE/SA/PVA.

The FT-IR examined all scaffolds to identify their cross-linkers throughout their functional groups [31]. Figure 2 shows the FTIR spectra of MOE/SA/PVA. The broad absorption bands between 3000 and 3700 cm^−1^ were linked to the stretching vibration of the O–H bond from inter- to intra-molecular hydrogen bonding in PVA and SA [32]. A reduction in the intensity of this absorption of these O–H bands in the scaffolds was observed by increasing the *Moringa oleifera* leaves extract, probably due to the interaction of the extracted constituent with the pendant hydroxyl groups of the polymer chains. These findings confirm the interaction of the extract components with the SA/PVA matrix. The sharp peak with weak intensity observed between 2800 and 3000 cm^−1^ can be attributed to the stretching vibration of C–H from alkyl groups. The scaffold’s peak at 1735 cm^−1^ was due to C=O from residual acetate groups in PVA and ester linkage [33,34]. This confirmed the extract functional groups’ interaction with the SA/PVA matrix. Further, a peak at about 1665 cm^−1^ corresponded to the hydroxyl bending mode [35]. This band was weakened, indicating the involvement of hydroxyl groups in scaffold formation and supporting the interactions between functional groups. The CH_2_ scissoring vibrations characteristic of PVA could be seen at 1458 cm^−1^ [36,37]. The bands at approximately 1033 cm^−1^ could be attributed to a strong C–O stretching region as a complex band resulting from C-O and C-O-C stretching vibrations. The peak that appeared for a scaffold without extract (S1) at 841 cm^−1^ corresponding to C−H bending had almost weakened, which provides evidence of the cross-linking between hydroxyl groups of extracted and SA/PVA matrix [38].

#### 3.2.2. X-ray Diffraction

**Figure 3:**
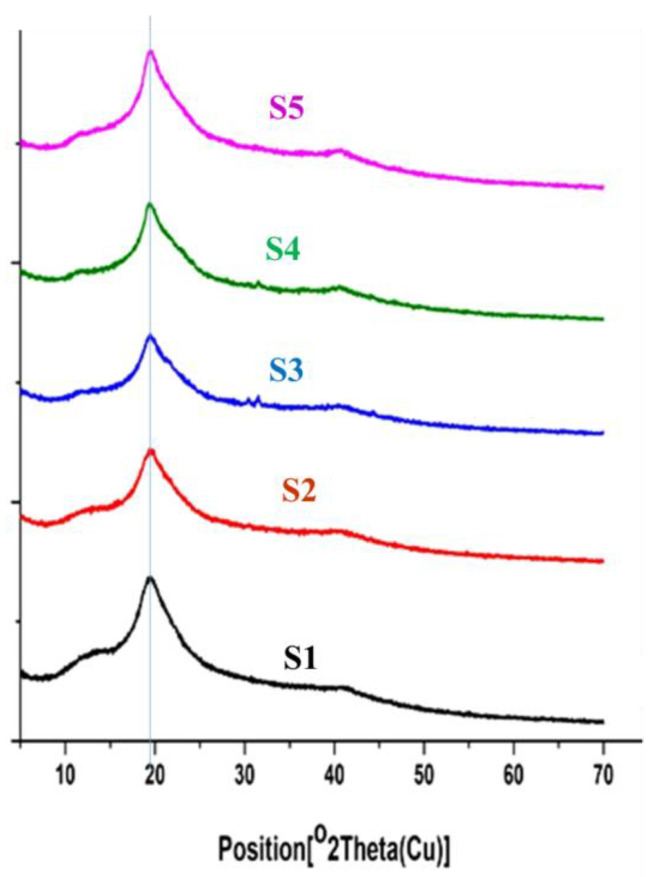
X-ray diffraction patterns of the MOE/SA/PVA.

The X-ray diffraction (XRD) patterns of the pure SA/PVA scaffold and MOE/SA/PVA are given in Figure 3. The pure SA/PVA scaffold and MOE/SA/PVA displayed an intense diffraction ray at 2θ~19.5° due to the strong hydrogen-bonding interactions [38,39]. These confirmed the homophonous dispersion of MOE during gelling. Moreover, weak XRD peaks at 2θ~40° proved a strong interaction between SA/PVA and MOE [40].

#### 3.2.3. Thermogravimetric Analysis

**Figure 4:**
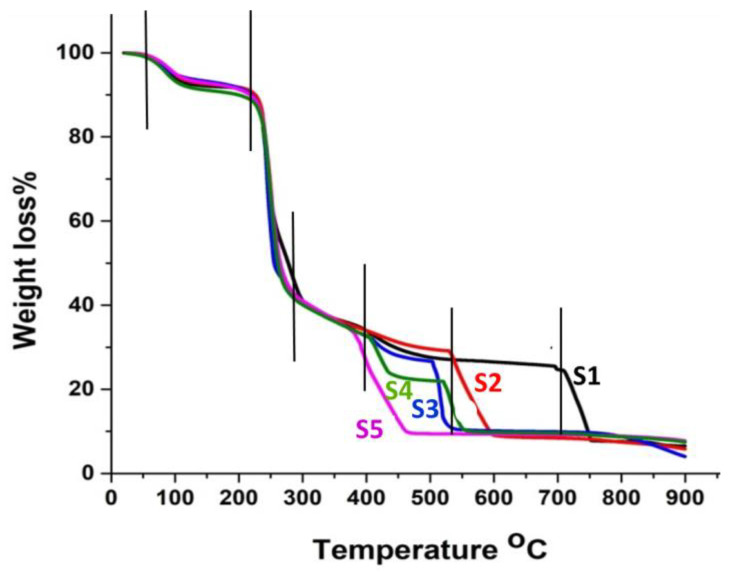
Thermogravimetric curve of the MOE/SA/PVA with a heating rate of 10 °C/min and under air atmosphere.

The thermal properties of the SA/PVA/*Moringa oleifera* leaves extracts were investigated by thermogravimetry. Figure 4 shows TG curves of the pure and extracts-immobilized scaffolds. As seen in the TG curves of the scaffolds, they were thermally broken down into three degradation stages. The first stage (approximately 10% weight loss) was observed between 25 and 100 °C due to the removal of physically bound water from the hydrogels. The second stage was detected at 100–280 °C (approximately 65% weight loss). This degradation step could be attributed to chain scission, a degradation of the main polymer chain, and the removal of water molecules.

These two steps were comparable for all samples, which referred to the thermal stability of the extracts in the scaffold up to 380 °C and confirmed the interaction between the extracts and the SA/PVA matrix through hydrogen bond formation. The last step was observed at 750, 600, 380, 450 and 460 °C for samples 1, 2, 3, 4 and 5, respectively (around 20% weight loss) because of carbonization [41]. The pure SA/PVA scaffold S1 exhibited a higher thermal stability compared to the scaffolds S2–S5, containing MOE. In more detail, for this step scaffold S1 showed 75% and 90% weight loss at 697 °C and 747 °C, respectively. The scaffolds S2, S3, and S4 forfeited 75% of their weight at 542, 506, and 404 °C and reached 10% residual weight at 593, 530, and 462 °C, respectively, while S5 forfeited 90% of its weight at 450 °C and the residual weight was 10% up to 900 °C. During this stage, weight loss occurred due to the decomposition, degradation, and vaporization of the stable polymer chain that remained.

#### 3.2.4. Morphological Study

**Figure 5:**
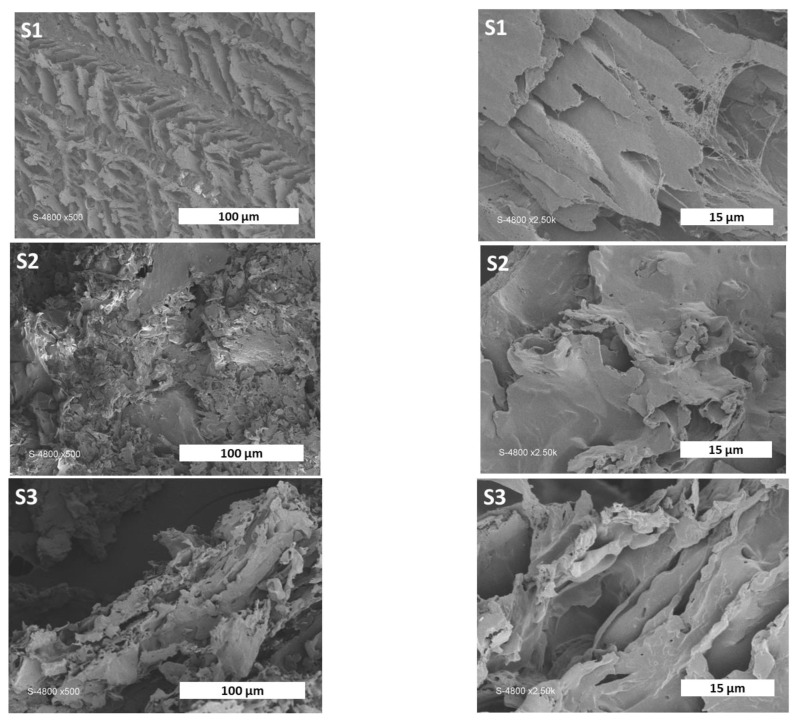

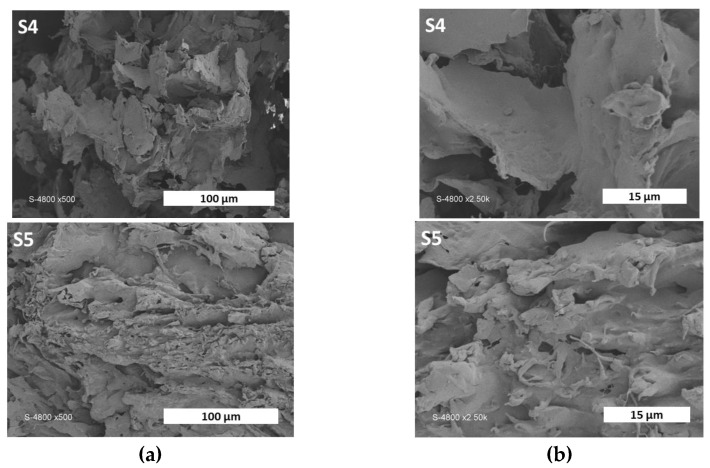
(**a**) SEM images of MOE/SA/PVA S1–S5 (scale bars: 100 µm), (**b**) SEM images with high magnification (scale bars: 15 µm).

Finally, the morphology of the materials was investigated via scanning electron microscopy (SEM). The SEM images of the materials S1–S5 are given in Figure 5. All materials showed lamellar architectures with interconnected porosity on the micrometric level. All materials showed homogeneous morphologies, suggesting that the components were homogeneous on the micrometric level. The lamellar morphology probably resulted from the freeze-drying process. Similar morphologies have already been observed for related PVA/cellulose composites [42]. It has to be pointed out that no incidence related to the increasing content of the MOE could be detected. The increasing MOE content therefore had no impact on the morphology of the samples.

### 3.3. Cytotoxicity Study

**Figure 6:**
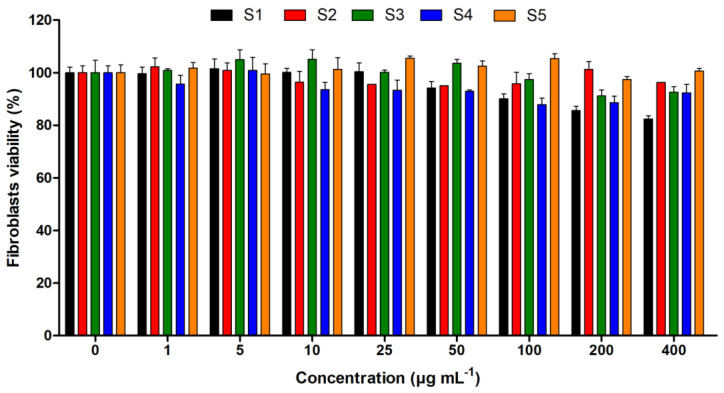
Cytotoxicity study of pure extract and MOE/SA/PVA on living human fibroblasts treated with different concentrations for 72 h. Results are presented as mean ± SEM (*n* = 3).

The results presented in Figure 6 show the low toxicity of pure and extract-immobilized SA/PVA scaffolds on human living fibroblasts. At 400 µg/mL, the cell viability values were above 90% for S2, S3, S4 and S5. However, for S1, the pure SA/PVA scaffold, the value was 82 ± 2%. The increase in cell viability can be explained by the beneficial properties of Moringa extract, which was present in increasing amounts in the formulations. These cytotoxicity results indicate the biocompatibility of MOE/SA/PVA.

### 3.4. In Vitro Wound Healing Studies

**Figure 7:**
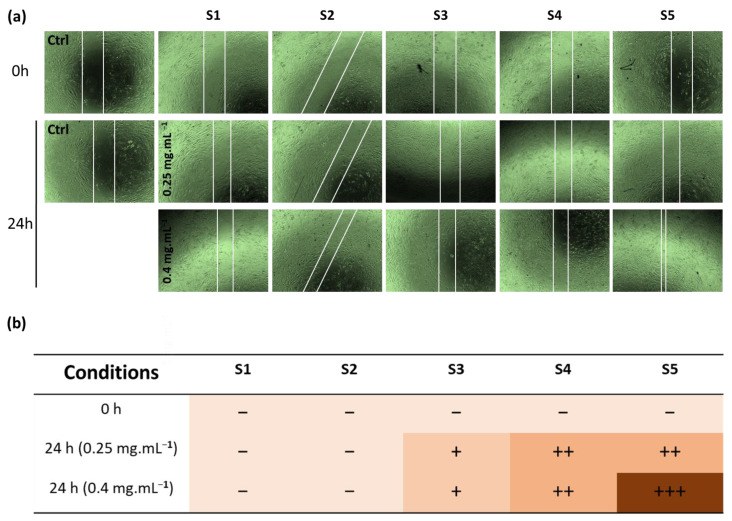
Cell scratch assay on human fibroblasts treated (or not) with different concentrations of pure extract and MOE/SA/PVA and observed at 0 to 24 h after treatment (**a**). Representative table of the wound-scratch-healing capacity of the different scaffolds when compared with the control (**b**).

In the present study, we performed cell scratch assays to observe the healing effect of the pure extract and MOE/SA/PVA on human fibroblasts treated with different concentrations (0, 0.25 and 0.4 mg/mL) for 0 and 24 h. As illustrated in Figure 7a, the scaffolds showed outstanding scratch closure capacity by migrating inwardly and covering bigger areas of the scratch than the control. The results demonstrated that the scaffolds enhanced the closure of cell scratches in a concentration-dependent manner, as represented in Figure 7b. Further, it is worth mentioning that the best healing activity was recorded for S5, which contained high MOE content, followed by S4 and then S3 scaffolds.

### 3.5. In Vivo Wound Healing Studies

**Figure 8:**
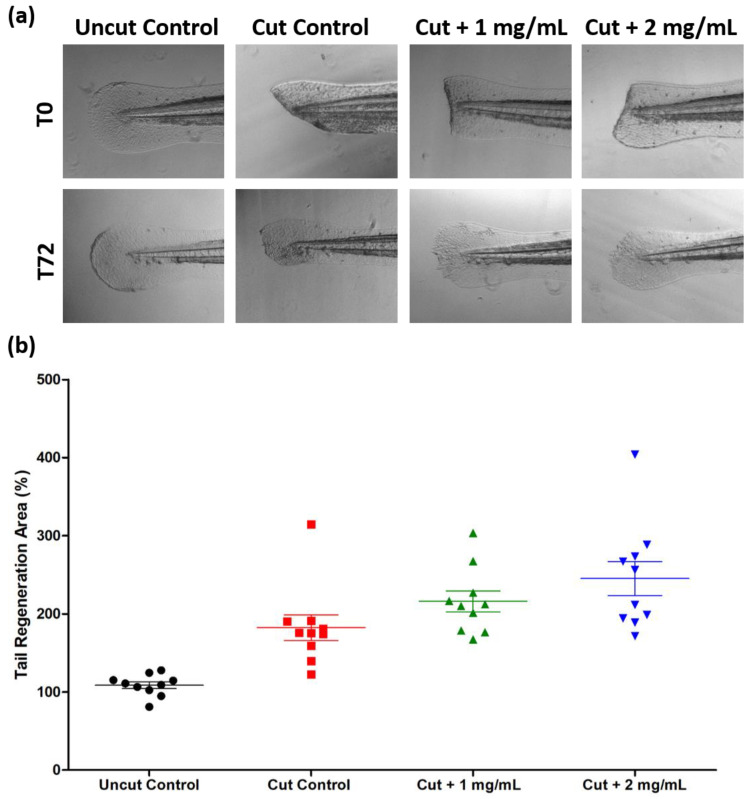
In vivo wound healing in zebrafish embryos. At 3 days post fertilization (dpf), zebrafish caudal tail fins were transected (or not), treated (or not) with 1 and 2 mg/mL of MOE/SA/PVA scaffold suspension (S5) and then images were taken at 0 to 72 h of regeneration after treatment using EVOS M5000 microscopy (**a**). Representative graph of the tail regeneration length capacity of the S5 scaffold when compared with the control (**b**).

The results presented in Figure 8 showed no change in the growth percentage for the uncut embryos after 3 days (T72); the value was 109 ± 14%. For the transected embryos, the growth percentage increased to 182 ± 51%. With the *Moringa oleifera*-extract-immobilized SA/PVA scaffolds (S5) treatment, increasing the concentration from 1 mg/mL to 2 mg/mL was associated with an increase in the growth percentage from 216 ± 42% to 246 ± 69%, respectively. These results showed the high capacity of *Moringa oleifera*-extract-immobilized SA/PVA scaffolds (S5) on wound healing in vivo.

## 4. Conclusions

In this work, natural polyphenolic flavonoids of *Moringa oleifera* leaves were extracted and immobilized into an SA/PVA matrix. The hydroalcoholic extract of MOE showed antioxidant activity and significant scavenging of free radicals. The scaffolds were successfully prepared using the freeze–thawing technique, and were characterized via FT-IR, XRD, SEM, and TG techniques. The FTIR spectra confirmed the hydrogen bond interaction between MOE and the SA/PVA matrix. Additionally, the immobilization of the *Moringa oleifera* leaves extracts decreased the thermal stability of the modified SA/PVA scaffolds. However, the incorporation of the extract had no impact on the morphology of the materials, as shown via scanning electron microscopy. The pure extract and MOE/SA/PVA showed higher biocompatibility to human fibroblasts up to 0.4 mg/mL concentrations. Further, the SA/PVA scaffolds containing a high content of MOE (S5) showed excellent wound healing capacity after 24 h of treatment at 0.4 mg/mL concentration. The S5 scaffold exhibited a good effectiveness for regenerating the caudal tail of zebrafish after transection.

## Figures and Tables

**Table 1:** TPC, TFC, and antioxidant activities of extracted *Moringa oleifera* leaves with different solvents.

Treatments	TPC mg/g	TFC mg/g	IC_50_ DPPH mg/mL
0.1 N K₂S₂O₈	70.8 ± 0.6 ^g^	1.3 ± 0.1 ^f^	3.12 ± 0.02
0.25 N NaOH	334.3 ± 2 ^a^	6.6 ± 0.2 ^d^	4.19 ± 0.03
0.25 N HCl	91.7 ± 0.8 ^f^	5.3 ± 0.1 ^e^	0.82 ± 0.01
80% Methanol	219.2 ± 1.6 ^b^	13.7 ± 0.4 ^c^	0.81 ± 0.01
80% Ethanol	182.8 ± 1.3 ^d^	19.9 ± 0.5 ^b^	1.01 ± 0.02
80% Ethylacetate	121.3 ± 1.2 ^e^	6.1 ± 0.2 ^d^	3.27 ± 0.04
80% Acetone	197.1 ± 1.5 ^c^	34.9 ± 0.6 ^a^	0.91 ± 0.01
5% LSD	2.2811	1.2193	---

Results are mean values of three replicates ± standard deviation. Different letter(s) in the same column indicates significant differences at *p* < 0.05.

**Table 2:** Polyphenolic compounds identified in the methanol extract of *Moringa oleifera* leaves.

Compounds	Conc. (µg/g)
Chlorogenic acid	22.5
Gallic acid	1.8
Caffeic acid	4.4
Rutin	0.6
Coumaric acid	150.4
Vanillin	4.5
Naringenin	269.8
Querectin	0.8
3,4-Dihydroxybenzoic acid	33.0
Cinnamic acid	22.0
Kaempferol	1.6
Ferulic acid	20.4
Syringic acid	1.9
Ellagic acid, Daidzein, Hesperetin, Myricetin, Methyl gallate, Apigenin, Catechin and Luteolin	ND

## Data Availability

Data are available on request.
